# Navigating the Zone of Injury: A Scoping Review of Outcomes Surrounding External Fixator Pin Placement in Open Fractures

**DOI:** 10.7759/cureus.97676

**Published:** 2025-11-24

**Authors:** Matthew Allen, Joseph Dale, Benjamin Kent

**Affiliations:** 1 Trauma and Orthopaedics, University Hospitals Plymouth NHS Trust, Plymouth, GBR; 2 Medicine, Worcestershire Acute Hospitals NHS Trust, Worcester, GBR

**Keywords:** external fixation, infection, open fractures, pin placement, scoping review, zone of injury

## Abstract

The anatomical placement of external fixator pins is considered critical in the management of open fractures, particularly with respect to avoiding compromised soft tissue. Although conventional surgical practices advise placing pins outside the zone of injury, this practice is based on expert opinion rather than peer-reviewed evidence. This gap poses potential adverse patient outcomes and warrants thorough clinical investigation. This scoping review aims to map the available literature on external fixator pin placement in relation to the wound zone of open fractures and identify any associated complications whilst examining current surgical guidance on this issue.

A scoping review was conducted in accordance with Preferred Reporting Items for Systematic Reviews and Meta-Analyses extension for Scoping Reviews (PRISMA-ScR) guidelines. Four databases (PubMed, EMBASE, Ovid MEDLINE, and Google Scholar) were searched, alongside grey literature sources such as the Arbeitsgemeinschaft für Osteosynthesefragen (AO) Surgery Reference and Orthopaedic Trauma Association (OTA) guidelines. Eligible sources included those addressing pin placement in open fractures, particularly in or near soft tissue wounds.

Of the 241 records screened, no peer-reviewed studies directly examined pin placement within or near the wound zone. A small number of studies discussed pin-plate overlap, with mixed findings on infection risk. Expert guidance from surgical references consistently recommends avoiding compromised soft tissue, but this is not supported by empirical evidence.

No clinical studies directly evaluate the outcomes of external fixator pin placement within open fracture wounds or compromised tissues. Existing guidance is largely based on expert consensus. Further research is needed to determine whether such placement impacts infection risk or clinical outcomes.

## Introduction and background

External fixation (ex-fix) is a technique of fracture immobilisation used in the initial and definitive management of open fractures, especially in cases involving significant soft tissue injury [1]. The strategic placement of external fixator pins is an important consideration to minimise complications such as infection, non-union, and neurovascular injury. Traditional surgical guidance often advises avoiding the placement of pins within the zone of injury or near open wounds, aiming to reduce the risk of contamination and interference with remaining periosteal blood supply [2,3].

Despite these recommendations, there is a notable lack of empirical evidence directly examining the outcomes associated with pin placement within or near the wound zone in open fractures. Most available recommendations derive from surgical textbooks, expert consensus, or technical manuals rather than from comparative or observational studies. There are instances within trauma surgery and the initial management of open fractures where the extent of soft tissue damage challenges conventional ideas regarding ex-fix pin placement. This can occur where there is extensive soft tissue damage or non-conventional fracture patterns, meaning large fragments appropriate for pin placement are adjacent to areas of compromised soft tissue. In some cases, this can necessitate placing ex-fix pins in proximity to, or even within, damaged soft tissue. This practice is contrary to advice from textbooks and expert consensus [2-5].

Given the potential clinical implications, a scoping review was undertaken to systematically map the available literature related to external fixator pin placement in the context of open fractures. This review aimed to identify studies discussing the anatomical placement of external fixator pins in relation to open wounds, examine reported complications such as infection when pins are placed in or near open wounds, and document the extent and type of guidance provided in surgical reference literature.

## Review

Methods

This scoping review was conducted in accordance with the Preferred Reporting Items for Systematic Reviews and Meta-Analyses Extension for Scoping Reviews (PRISMA-ScR) guidelines [6]. We considered all publications, research articles, guidelines, case reports, and grey literature that commented on the anatomical location of ex-fix pin placement in open fractures. Sources addressing ex-fix pin location, wounds, and infection rates relating to pin placement were considered, regardless of study design. Grey literature sources were included on account of the absence of primary studies in this field. Only English language sources were included owing to resource constraints regarding translation. Studies focused solely on closed fractures (such as in the pelvis), complications of ex-fix unrelated to pin placement, or with no comment on anatomy were excluded. Cadaveric studies were also excluded.

Search strategy

Electronic databases PubMed, Ovid MEDLINE, Ovid EMBASE, and Google Scholar were searched. In addition, grey literature sources such as the Arbeitsgemeinschaft für Osteosynthesefragen (AO) Surgery Reference and the Orthopaedic Trauma Association (OTA) guidelines were included. A comprehensive search strategy was developed using a combination of medical subject headings (MeSH) and free-text keywords to identify relevant literature on ex-fix in the context of open fractures. The search targeted core concepts, including ex-fix, open fractures, pin placement, anatomical safe zones, and complications such as infection. Search strategies were tailored to match the syntax and controlled vocabulary of each database.

In PubMed, the initial search combined MeSH terms and synonyms using the following Boolean structure: ("external fixation"[MeSH Terms] OR "external fixator" OR "external fixation") AND ("open fracture"[MeSH Terms] OR "open fractures" OR "compound fracture") AND ("safe zone" OR "zone of injury" OR "injury zone" OR "soft tissue envelope") AND ("pin" OR "pin site" OR "pin placement" OR "half-pin" OR "Schanz screw") AND ("infection" OR "complication" OR "pin tract infection" OR "pin-site infection"). This strategy yielded four results, none of which were directly relevant to the review question. To increase sensitivity, a revised search was performed using broader anatomical descriptors in place of “safe zone” terms. The updated strategy included the following: ("external fixation"[MeSH Terms] OR "external fixator" OR "external fixation") AND ("open fracture"[MeSH Terms] OR "open fractures" OR "compound fracture") AND ("pin placement" OR "pin site" OR "half-pin" OR "Schanz screw") AND ("soft tissue" OR "anatomical location" OR "proximal placement" OR "pin location") AND ("infection" OR "complications" OR "pin tract infection"). This produced 16 results, of which only one was deemed indirectly relevant. Given the low number of directly applicable results, a broader free-text search using the terms “external fixator AND pin placement” was also conducted. This approach returned 130 results on PubMed, many of which were unrelated to the desired topic; a small number of papers included themes such as pin site overlap with definitive metalwork. These papers were considered indirectly relevant.

Selection of sources

**Figure 1:**
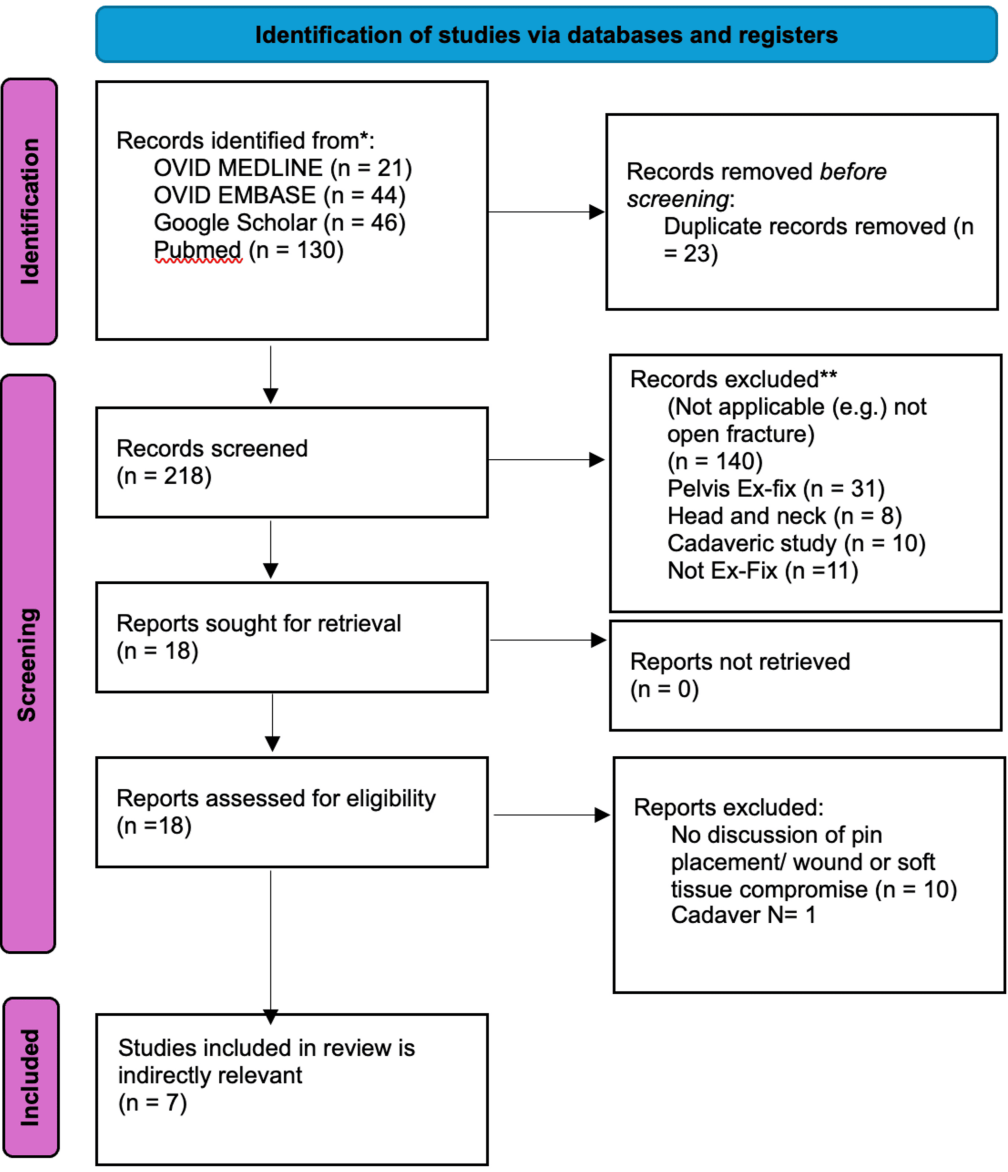
PRISMA flow diagram PRISMA: Preferred Reporting Items for Systematic Reviews and Meta-Analyses, Ex-fix: External fixation

After removal of duplicates, titles and abstracts were screened by two independent reviewers. Full-text screening was performed for articles as mentioned that were indirectly relevant to the inclusion criteria. Any disagreements were resolved through discussion. The selection process was documented using a PRISMA flow diagram (Figure 1) [7].

Results

A total of 241 records were identified through searches of four databases: PubMed (n=130), EMBASE (n=44), Ovid MEDLINE (n=21), and Google Scholar (n=46). Following the removal of duplicates, 218 unique records were screened by title and abstract. Of these, 19 records were selected for full-text review. No peer-reviewed studies met the predefined eligibility criteria for inclusion in this scoping review. 

While no studies directly examined the outcomes of external fixator pin placement within or near the wound zone of open fractures, several articles were identified that discussed related topics. In particular, a small group of studies explored the relationship between pin site overlap and subsequent definitive fixation, such as plates or intramedullary nails. None of these studies made specific reference to open wounds, wound zones, or areas of soft tissue compromise when discussing pin placement strategies.

To address the lack of direct evidence, relevant surgical reference materials and guidelines were reviewed. The AO Surgery Reference provides detailed anatomical guidance for pin insertion, emphasizing the avoidance of areas with extensive soft tissue damage or fracture hematoma. It also advises planning pin placement with consideration for future definitive fixation [8]. Similarly, the OTA principles of ex-fix recommend placing pins outside the zone of compromised skin, typically described as the 'zone of injury,' and within recognised anatomical safe corridors [3,9]. While these documents offer practical guidance, they are based on expert consensus and anatomical principles rather than clinical outcome data.

In the peer-reviewed literature, our search found five studies examining the relationship between overlapping ex-fix pin sites and definitive fixation. The studies reviewed yielded conflicting evidence. A 2012 study by Laible et al. showed no statistical significance between pin-plate overlap and infection rates of high-energy tibial plateau fractures [10]. This was a small retrospective cohort study of 79 and included only six infections; thus, all drawn conclusions should consider the potential underpowered nature of these results. A retrospective cohort study of 244 patients by Haase et al. in 2022 suggested that pin-plate overlap in tibial plateau fractures was associated with increased infection rates. This is a statistically significant and seemingly robust conclusion, as multivariate binary logistic regression confirmed pin-plate overlap as an independent risk factor for the development of deep infection [9]. In contrast, a larger retrospective study by Potter et al. that included 277 patients with Pilon fractures reported no statistically significant association between pin site overlap and postoperative infection, nor did it find any relationship with the duration of external fixator application [11]. Similarly, a 2019 study by Hadeed et al. involving 178 patients concluded that pin overlap did not significantly affect infection rates [12]. Collectively, these studies suggest that while overlap may influence outcomes in specific anatomical regions, such as the tibial plateau, the overall evidence is inconclusive. None of the studies evaluated pin placement with respect to the wound zone or soft tissue integrity.

One article from the American Academy of Orthopaedic Surgeons discussed principles of pin placement, recommending that pins be positioned as close as possible to the fracture site, yet outside the fracture line, to optimise construct stiffness. The article also referenced the use of ex-fix in humeral fractures with open wounds, but it provided no guidance regarding the avoidance of open or threatened soft tissue zones, beyond standard precautions to avoid neurovascular structures [5].

Additionally, a publication describing a consensus survey of 255 active members of the OTA provided insight into common practices in lower extremity ex-fix. This study, based on responses to a 40-question protocol-focused questionnaire, emphasised the importance of CT imaging both pre- and post-external fixator application. It also highlights limb length maintenance and avoidance of pin site overlap with definitive fixation as key priorities in temporary fixation planning. However, as with other sources, the survey did not address pin placement in relation to open wounds or compromised soft tissue [4].

Discussion

To our knowledge, this is the first paper to systematically review the literature surrounding complications such as infection rates associated with ex-fix pin placement within or in proximity to wounds in the management of open fractures. This scoping review aimed to identify and synthesize available evidence regarding the placement of external fixator pins in relation to the wound zone in open fractures. Despite an extensive search of peer-reviewed literature and grey sources, no studies were identified that directly investigated clinical outcomes associated with placing pins within or adjacent to areas of soft tissue injury. This represents a notable gap in the existing literature.

Current practice is informed predominantly by surgical guidelines and technical manuals rather than clinical studies. Resources such as the AO Surgery Reference and OTA guidelines provide detailed anatomical instructions for pin placement, often recommending avoidance of the fracture, hematomas, and compromised soft tissue regions. These recommendations are thought to be based on anatomical, biomechanical, and physiological reasoning but are not supported by comparative data. Terms such as "safe zones" and "zone of injury" are used in these recommendations but lack standardised definitions or outcome validation in the published literature.

The lack of empirical evidence is particularly significant given the clinical importance of pin placement in open fracture management. Surgeons routinely aim to minimise infection risk and optimise the outcomes of definitive fixation, yet no studies have evaluated whether pin placement in or near the wound site will affect outcomes. While a small number of studies have investigated the consequences of ex-fix pin overlap with definitive fixation hardware, their relevance to outcomes surrounding the placement of ex-fix pins remains limited [9-12]. The findings from these studies are inconsistent, and none directly address the influence of soft tissue compromise on pin-site complications.

Limitations

This scoping review has some notable limitations. Although a comprehensive and systematic search strategy was applied across major databases, including PubMed, EMBASE, Ovid MEDLINE, and Google Scholar, there remains a risk that relevant studies were missed due to inconsistent or non-standardised terminology used in the literature. Terms such as “safe zone,” “zone of injury,” or “wound site” are not uniformly defined across publications, which may have limited retrieval. Grey literature sources, including surgical manuals and expert guidelines, were incorporated to address the evidence gap; however, these materials are not peer-reviewed and often reflect expert opinion rather than evidence-based findings. Their inclusion was considered necessary due to the paucity of formal studies on the topic. Lastly, the review was restricted to English-language sources, potentially excluding relevant work published in other languages, particularly from regions with high volumes of trauma surgery where different practices may exist.

## Conclusions

This review highlights a clear opportunity for further research. Prospective observational studies and retrospective cohort analyses are needed to evaluate whether pin location relative to the wound zone influences outcomes such as infection. Given the widespread use of ex-fix and the potential consequences of suboptimal pin placement, establishing an evidence base in this area is both necessary and needed in a timely fashion.

## References

[1] Hadeed A, Werntz RL, Varacallo MA (2023). External Fixation Principles and Overview. StatPearls [Internet]. 2023 Aug 4 [cited.

[2] Höntzsch D, Disclaimer SB (2025). AO Trauma: principles of external fixation. https://tinyurl.com/ys5bbxpz.

[3] Khokhar R. Core Curriculum V5 PRINCIPLES OF EXTERNAL FIXATION (2025). Orthopaedic Trauma Association principles of external fixation. Orthopaedic Trauma Association.

[4] Collinge C, Kennedy J, Schmidt A (2010). Temporizing external fixation of the lower extremity: a survey of the orthopaedic trauma association membership. Orthopedics.

[5] Bible JE, Mir HR (2015). External fixation: principles and applications. J Am Acad Orthop Surg.

[6] Page MJ, McKenzie JE, Bossuyt PM (2021). The PRISMA 2020 statement: an updated guideline for reporting systematic reviews. BMJ.

[7] (2025). PRISMA 2020 flow diagram — PRISMA statement. https://www.prisma-statement.org/prisma-2020-flow-diagram.

[8] (2025). Modular external fixation. https://surgeryreference.aofoundation.org/orthopedic-trauma/adult-trauma/basic-technique/basic-technique-modular-external-fixation.

[9] Haase LR, Haase DR, Moon TJ (2022). Is pin-plate overlap in tibial plateau fractures associated with increased infection rates?. Injury.

[10] Laible C, Earl-Royal E, Davidovitch R, Walsh M, Egol KA (2012). Infection after spanning external fixation for high-energy tibial plateau fractures: is pin site-plate overlap a problem?. J Orthop Trauma.

[11] Potter JM, van der Vliet QM, Esposito JG, McTague MF, Weaver M, Heng M (2019). Is the proximity of external fixator pins to eventual definitive fixation implants related to the risk of deep infection in the staged management of tibial pilon fractures?. Injury.

[12] Hadeed MM, Evans CL, Werner BC, Novicoff WM, Weiss DB (2019). Does external fixator pin site distance from definitive implant affect infection rate in pilon fractures?. Injury.

